# Depressive symptoms, HIV-related stigma and ART adherence among caregivers of children in vulnerable households in rural southern Malawi

**DOI:** 10.1371/journal.pone.0247974

**Published:** 2021-03-05

**Authors:** Kathryn L. Spielman, Erica Soler-Hampejsek, Adamson S. Muula, Lyson Tenthani, Paul C. Hewett

**Affiliations:** 1 Population Council, Washington, DC, United State of America; 2 Independent consultant, Barcelona, Spain; 3 University of Malawi, College of Medicine, Blantyre, Malawi; 4 ICAP Malawi, Lilongwe, Malawi; National University of Singapore, SINGAPORE

## Abstract

**Background:**

Few studies have explored the association between depressive symptoms, HIV infection and stigma in vulnerable populations. The objective of this study is to examine factors associated with depressive symptoms among caregivers living in vulnerable households in Malawi and assess how reported depressive symptoms and other factors affect ART adherence among caregivers who report testing positive for HIV and currently on ART.

**Methods:**

We interviewed 818 adult caregivers of children aged 0–17 years living in vulnerable households in 24 health facility catchment areas in five districts in rural southern Malawi in 2016–2017. Vulnerable households had either economic and food insecurity, or chronic illness. Questions on five depressive symptoms were used. ART adherence was self-report of not forgetting to take ART medication in the last week. Perceived and anticipated measures of stigma were used. Multivariable linear and logistic regressions documented relationships between depressive symptoms, self-reported HIV status, HIV-related stigma, and ART adherence.

**Results:**

Most caregivers were women (86.2%); about one third had no spouse or live-in partner. Fifty-seven percent of caregivers reported having three or more depressive symptoms. Forty-one percent of caregivers reported testing positive for HIV. Self-reported HIV positive status was associated with depressive symptoms (adjusted coeff = 0.355, p-value <0.001), which were in turn associated with poorer ART adherence among caregivers (aOR 0.639, p-value = 0.023). HIV-related stigma was also associated with depressive symptoms for caregivers who reported having HIV (coeff = 0.302, p-value = 0.028) and those who reported testing negative for HIV (coeff = 0.187, p-value <0.001). Having social support was associated with lower depressive symptoms (coeff = -0.115, p = 0.007). HIV-related stigma, having social support, and other socio-demographic characteristics were not found to be associated with ART adherence.

**Conclusions:**

Addressing mental health among caregivers in vulnerable households may be an important step toward achieving viral suppression among vulnerable populations living with HIV in Malawi. Integrating depression screening into HIV care and treatment protocols could be a promising intervention to improve longer-term outcomes.

## Background

Mental health disorders, such as depression, and human immunodeficiency virus (HIV) have been found to be intertwined. Mental health issues are associated with risk factors for HIV infection [1] and HIV positive status [2]. Evidence suggests that HIV-related stigma can be a cause of negative mental health outcomes. Individuals who have tested positive for HIV may have chronically high stress levels due to experiencing stigma [3]. HIV-stigma has been linked to greater loneliness and social isolation, and it is consistently associated with depression and depressive symptoms [4]. For those who have not been tested, stigma has been found to be a barrier to getting tested, as individuals want to avoid being seen at a testing site and/or fear the negative consequences of a positive test result [5]. Depression may be related to HIV disease progression—depressive symptoms may be associated with antiretroviral therapy (ART) nonadherence, even at levels below the cutoff for clinical depression [6,7].

A growing body of research is beginning to document the prevalence of depression and links between HIV and depression in Malawi, a country that has limited infrastructure and resources to address mental health. A recent study in Malawian primary care settings found that when screening is implemented about 2 out of 10 of all patients, regardless of HIV status, have depression [8], and depression levels have been found to be similar among certain populations living with HIV, including adolescents in HIV clinics [9] and adults on ART [10].

Despite evidence of depression among adults living with HIV in Malawi, there has been little research on depression among caregivers of children living in vulnerable households in communities with high HIV-prevalence. The physical and psychological toll of HIV affects not only those infected, but also their relatives, household members, and neighbors. Children may be orphaned due to HIV and AIDS, or under the care of parents who do not have their full strength for caregiving. For families living in HIV-endemic communities, the burden of caregiving for sick family members and orphaned and other vulnerable children can be significant [11]. Caregivers of orphaned and vulnerable children can be vulnerable themselves, often living in difficult socio-economic conditions [12] and caring for household members with chronic illnesses, such as HIV and AIDS, if not being chronically ill themselves. Caregivers of children in HIV-affected families have been found to display depressive symptoms in a variety of settings across the globe [13–15]. The wellbeing of caregivers and their ability to cope with stressors in their lives affects, in turn, the wellbeing of the children in their care [16–18]. Strong social support networks can help buffer these negative effects and promote good mental health outcomes despite these burdens [19].

To tailor interventions to improve caregiver and child wellbeing in Malawi, links between depression and HIV must be examined. While other studies in Malawi have explored this relationship, their samples have comprised women [20], mothers of young infants [21], adolescents [22], or adults living with HIV [23]. To our knowledge, no other study has examined prevalence and correlates of depressive symptoms among primary caregivers of children in vulnerable households in Malawi. In light of this, the aim of this study was to examine depressive symptoms among a representative sample of caregivers of children in vulnerable households and explore relationships with self-reported HIV status, HIV-related stigma and, for caregivers who reported testing positive for HIV and on treatment, ART adherence.

## Data and methods

### Sampling and participants

A representative sample of 818 adult caregivers of children in vulnerable households within five rural districts in the southern region of Malawi–Blantyre, Chikwawa, Mangochi, Mulanje, and Phalombe–were recruited to participate in a cross-sectional survey interview in December 2016 to April 2017. The districts were selected and data were collected as part of a study aimed at monitoring a USAID-supported program to reduce new HIV infections and alleviate the impact of HIV among at-risk populations. The data we use in this paper derive from the first of three yearly rounds of data collection, before the program had expanded to the study areas.

Twenty-four health facilities were purposely selected in the five districts, and a 5-kilometer (km) radius was mapped around each facility as a catchment area proxy. Three census enumeration areas (CEAs) were randomly selected within each selected catchment area. A household screening was conducted in all 72 CEAs between December 2016 and January 2017 to identify households that fit the eligibility criteria. i.e., vulnerable households with children aged 0–17 years and adult caregivers.

Household vulnerability was defined as having either: 1) both economic and food insecurity, or 2) chronic illness in the household, as reported by the household head at the household screening. A household was considered economically insecure if the household head reported that the household was not able to pay for an unexpected household expense (e.g. emergency medical expense including transport to a facility or house repair) on the day of the screening, if the household head or spouse reported any form of disability or illness that prevented him/her from engaging in work, or if no member in the household had consistent income generating work in the 6 months prior to the household screening. Food insecurity existed if, within the previous four weeks, there was ever an instance where there was no food to eat of any kind in the household because of lack of resources to get food, a household member recently went to sleep hungry or a household member recently went a whole day and night without eating. Chronic illness was established in cases where the household had one or more members who the household head reported to be living with HIV, the household had one or more adult member/s who had been very sick for at least three months during the past year, or the household had one or more member/s who the household head reported was taking long-term medication.

The original sample size was generated for the purposes of conducting a stepped-wedge randomized evaluation. To determine the required sample size of participants for the planned impact evaluation, a Stata 13.0 routine sample size for stepped-wedge cluster randomized trials was used to detect a minimal 10% percentage point effect size between an intervention and control group in key indicators. The sample was also inflated to account for non-response and clustering.

Vulnerable households with children aged 0–17 were identified from the household screening and a sample of 863 households was randomly selected across the 72 CEAs to participate in the round 1 survey. Of the 863 households, 818 completed the survey (95% completion) during March to April 2017, with the most common reasons for incompletion being that the household had moved from the CEA (2%) or the eligible respondent was not present and would not return within two weeks (2%). In households with more than one eligible child, one child was randomly selected. A primary adult caregiver of the child was selected for the interview; if he/she was unavailable, a secondary adult caregiver was selected.

### Survey instrument

The survey instrument included an extensive set of questions regarding both the caregiver him/herself and about one child in the household. All questions were answered by the caregiver. Questions regarding the caregiver included: basic demographics, household wealth, self-reported HIV testing experience and status, HIV stigma, sexual behavior, attitudes toward violence, gender/social norms, social support and psychosocial wellbeing, perceptions around community social cohesion and collective efficacy; specific measures are discussed in the next section. Questions regarding the child included relationship to caregiver, information on biological parents, education and stimulation (child’s engagement in physical and mental activities), chores, work or other activities, health and psychosocial wellbeing, vaccinations, child HIV status and disclosure, food consumption and child discipline. S1 Table presents the survey items of each variable of interest. The survey includes questions adopted from multiple sources, such as the 2010 Malawi Demographic and Health Survey (DHS) [24], the Malawi 2014 UNICEF Multiple Indicator Cluster Surveys (MICS) [25], the 2013 Malawi Labor Force Survey [26], the Measure Evaluation Caregiver Questionnaire version 1.4, and orphan and vulnerable children (OVC) Questionnaire version 1.3 [27]. The survey was first developed in English. Translations to Chichewa and Yao were completed in collaboration with Wadonda Consult Limited. Ten days of classroom training with enumerators were held during late February to early March 2017, during which it was explained what each survey question was intending to ask, how to ask sensitive questions from respondents, and to ensure questions were culturally appropriate. Translations were revised based on feedback during the classroom training sessions. The survey was adopted to the local context during a pre-test in early March 2017 with a sample of 103 respondents from a similar setting to the study setting. The pre-test location was in South Lunzu in Blantyre district. All data were collected electronically using tablet computers with the interviewer asking all questions and entering responses.

### Ethical approval

Written consent was obtained from each participant. Participants who could not read or write could mark consent forms with a fingerprint. All interviews were conducted in where privacy could be maintained during the interview. Data was stored electronically and encrypted and secured on devices protected by passwords. All informed consent and assent forms were kept in locked file cabinets in a study office and only research staff had access to these forms. The household screening, survey methods and procedures were reviewed and approved by the University of Malawi College of Medicine Research and Ethics Committee (P.11/16/2067 and P.09/16/2019), as well as the Population Council Institutional Review Board (p772 and p785).

### Measures

#### Depressive symptoms

Five items were adapted from the Child Status Index [28] and included in the survey both as questions about the child and about the caregiver to assess depressive symptoms. Four of the five items are similar to items from the 9-item Patient Health Questionnaire (PHQ-9), a validated screening tool for depression in adults [29]. The fifth item, which assessed irritability, was determined to be appropriate for inclusion as it was determined to be a symptom for major depressive disorder (MDD) in adults by Fava et al. [30] The measures of depressive symptoms were based on a four-week recall period. The self-reported depressive symptoms were: (1) taking little interest or pleasure in things typically enjoyed, (2) feeling down, depressed or hopeless, (3) having trouble falling or staying asleep, (4) sleeping too much or for too long, and (5) feeling irritable. The response options were scored 0 for never, 1 for sometimes, and 2 for often, for an unstandardized summative depressive symptom score range of 0–10. The total score was then standardized to have a mean of 0 and a standard deviation of 1. As a cutoff to diagnose depression has not been validated with these items, the score was standardized to represent a relative measure of depressive symptoms within the sample. The Cronbach’s alpha for the five depressive symptoms items was 0.74, denoting acceptable internal consistency.

#### Social support

To measure current social support, we asked caregivers about their emotional social support (has someone to turn to for suggestions with how to deal with a personal problem), tangible social support (has someone to help with daily chores if the caregiver is sick), and social companionship support (has someone who shows love and affection, has someone who is available to do something enjoyable with). The Cronbach’s alpha for the four social support items was 0.63, denoting moderately acceptable internal consistency. The responses to four items scored 0–1 were summed and the total score was standardized to have a mean of 0 and standard deviation of 1.

#### HIV-related stigma

HIV-related stigma was measured with items adapted from the Link-Up study in Uganda [31]. Perceived (external) stigma was assessed for caregivers who reported being HIV positive and had disclosed their status. A score was created using a count of the number of stigma experiences perceived. For those who reported either being HIV negative or never having been tested, an anticipated stigma score was created as a count of the number of experiences that they would expect to occur if they tested positive for HIV. Experiences for both anticipated and perceived stigma included loss of job/livelihood, friends, and significant other; rejection or neglect by family, coworkers, and community; difficulty finding sexual partners; negative experiences with health providers; and violence from spouse/partner. The total summative score was standardized to have a mean of 0 and standard deviation of 1. The Cronbach’s alpha for perceived stigma and anticipated stigma were 0.74 and 0.90 respectively, denoting acceptable internal consistency for the perceived stigma scale and strong internal consistency for the anticipated stigma scale.

#### ART adherence

Adherence to ART was assessed based on a question asking about the number of days the caregiver reported missing or forgetting to take their ART medication in the previous 7 days. Those who reported not missing any days were considered adherent.

### Analytical approach

To generate descriptive statistics for caregiver and household characteristics, we generated means and proportions adjusted for clustering at the CEA level using the svy command in Stata. We used linearized logistic and linear regressions adjusted for clustering at the CEA level to generate 95% confidence intervals (CIs) to indicate how precise the measures are given the sample size. Next, we ran regression analyses on two distinct samples. First, a multivariable linear regression was used to assess the relationship between the caregiver depressive symptoms outcome and economic security, food security, HIV status, HIV-related stigma and social support, as covariates. Variable selection was based on a review of the literature and all variables were retained in the models. The analytical sample for this model consisted of all caregivers, regardless of HIV testing history and reported HIV status, that had non-missing values for all variables of interest. This sample is henceforth referred to as the full analytical sample. Second, multivariable logistic regression was used to examine the association between depressive symptoms and ART adherence among caregivers who reported living with HIV and taking ART medication and had non-missing values for all variables of interest. This sample, which is a subset of the full sample, is henceforth called the ART adherence sample. The level of significance for regressions was 0.05. All regressions controlled for caregiver education, age, sex, marital status, household wealth, and standard errors were adjusted for clustering at the CEA level. Regressions for ART adherence also controlled for perceived HIV-stigma and social support. Household wealth was created using the Demographic and Health Survey method, including housing conditions (roof, wall, floor material), water source, and assets, and using polychoric principal component analysis to construct a wealth score that was subsequently split into quintiles [32]. Caregivers with missing responses for any relevant variable were excluded from analyses. Data were analyzed using Stata statistical software (version 15.1; STATA Corp, College Station, Texas).

## Results

### Who are caregivers in vulnerable households?

A total of 818 caregivers aged 18 or older completed the survey. The full analytical sample, i.e., those with responses on all variables of interest for the analysis, comprises 761 (93%) caregivers. Among caregivers excluded from the analysis due to missing responses, significantly fewer had ever tested for HIV and significantly more were food insecure compared to those in the full analytical sample. Table 1 displays demographic characteristics of the two analytical samples: both the full analytical sample and the subset of reported HIV positive caregivers included in the model for ART adherence (n = 289).

**Table 1:** Caregiver and household characteristics.

Caregivers (self-reported)	Full analytical sample (n = 761)		ART adherence sample (n = 289)	
	Proportion or mean	95% CI	Proportion or mean	95% CI
Female, %	86.2	(83.4, 88.6)	89.3	(85.2, 92.3)
Age, mean	40.7	(39.6, 41.9)	38.0	(37.0, 39.0)
Has completed primary or higher, %	15.1	(12.0, 18.9)	15.6	(11.4, 21.0)
No spouse or live-in partner, %	36.3	(32.4, 40.4)	44.6	(39.3, 50.1)
Number of children in care, mean	3.6	(3.5, 3.8)	3.6	(3.4, 3.8)
Relationship to child, %				
Biological son/daughter, %	71.1	(66.8, 75.1)	81.0	(75.8, 85.3)
Step/adopted son/daughter, %	2.8	(1.8, 4.2)	4.2	(2.3, 7.3)
Nephew/niece, %	4.2	(3.0, 6.0)	3.1	(1.5, 6.3)
Grandchild, %	19.3	(16.1, 23.1)	9.3	(6.6, 13.2)
Other relative, %	2.6	(1.6, 4.3)	2.4	(1.1, 5.5)
Ever tested for HIV, %	94.9	(93.2, 96.2)	100.0	-
Tested for HIV in past year, %	66.9	(61.9, 71.5)	72.2	(44.0, 90.0)
Living with HIV (self-reported), %	40.7	(37.2, 44.3)	100.0	-
Reported at least 1 depressive symptom, %	86.7	(83.8, 89.2)	86.9	(82.1, 90.5)
Reported at least 3 depressive symptoms, %	57.3	(52.7, 61.8)	60.2	(54.1, 66.0)
**Household (reported by household head in household screening)**				
Economically and food insecure, %	23.9	(19.9, 28.5)	8.7	(5.9, 12.5)
Economically insecure, %	60.7	(55.9, 65.3)	51.6	(44.6, 58.4)
Food insecure, %	30.5	(26.0, 35.4)	16.6	(12.2, 22.2)
Chronic illness in household, %	84.6	(81.0, 87.6)	100%	-
One or more adult member had been very sick for at least 3 months during the past year, %	42.8	(39.1, 46.7)	30.8%	(25.9, 36.1)
One or more member currently taking long-term medication, %	59.7	(55.2, 63.9)	94.1%	(91.1, 96.1)
One or more household member currently living with HIV, %	38.6	(35.3, 42.1)	100%	-

Among those in the full analytical sample, caregivers were a mean age of 40.7 years old, with a range from 18 to 89 years old, and 14% of caregivers were aged 60 and older. Highest level of schooling was low, with only 15% having completed primary school or more. Most caregivers were female and cared for a median of 3 children, including biological and non-biological children. About 1 in 3 caregivers were unmarried or not living with a partner. Almost all caregivers (95%) had ever been tested for HIV, and 41% of those tested reported a positive HIV test result. Excluding those caregivers who reported living with HIV for longer than one year, 67% were tested for HIV within the previous year. Caregivers included in the model for ART adherence have similar characteristics, although fewer had a spouse or live-in partner.

More than half of caregivers’ households were economically insecure, and about one third were food insecure. Twenty-four percent of caregivers’ households were both economically and food insecure. Living in more than 85% of the caregivers’ households was at least one adult who had been chronically ill for at least 3 months in the prior year. Caregivers in the ART adherence model sample have similar characteristics, except about half as many were food insecure compared to caregivers in the full analytical sample.

### Depressive symptoms

Depressive symptoms were high among caregivers, with 87% of those in the full analytical sample reporting at least one depressive symptom sometimes or often in the previous four weeks and 57% reporting three or more depressive symptoms sometimes or often in the previous four weeks. Among caregivers reporting a positive HIV test result and currently on ART, 87% reported at least one depressive symptom sometimes or often in the previous four weeks, and 60% reported at least three symptoms sometimes or often in the previous four weeks.

### HIV-related stigma

Approximately 41% of caregivers self-reported living with HIV. Table 2 presents frequencies for events that the caregiver reported either occurred (if reported to be HIV positive and disclosed to someone) or thought were likely to occur if they were to test positive for HIV and tell someone (for those who had tested negative for HIV or never tested). The average number of HIV-related stigma events perceived by caregivers who reported a positive HIV test result in this sample was 1.0 on a range of 0–10, with a standard deviation of 1.6. The average number of HIV-related stigma events anticipated by caregivers who had never been tested for HIV or who had tested negative was 2.5 (range 0–10, standard deviation 3.1). This average suggests low perceived and anticipated stigma in the setting. The most commonly reported experienced stigma events were difficulty finding sexual partners (23.5%), family not caring for her/him when sick (15.0%), community treating her/him like a social outcast (13.6%), and spouse/partner(s) becoming violent upon learning that she/he had the virus (12.2%). The most commonly reported anticipated stigma events were difficulty finding sexual partners (44.4% for those who reported testing HIV-negative, 25.6% for those who reported never being tested for HIV), experiencing break-up of marriage or relationship (28.7% for those who reported testing HIV-negative, 18.0% for those were who reported never being tested), and spouse/partner(s) becoming violent upon learning that she had the virus (25.0% for those who reported testing HIV-negative, 20.5% for those who reported never being tested). There was greater anticipated stigma among caregivers who had never been tested for HIV or had tested negative compared to experienced stigma among those who had tested as positive for HIV.

**Table 2:** HIV-related stigma among caregivers reporting to be living with HIV, reporting to be negative for HIV and never having been tested.

Scenario	Tested positive for HIV (n = 294) % (95% CI)	Tested negative for HIV (n = 428) % (95% CI)	Never tested for HIV (n = 39) % (95% CI)
Lost job/livelihood	4.8 (2.8, 7.9)	20.3 (16.6, 24.7)	28.2 (15.9, 44.9)
Treated badly at work or school	3.4 (1.8, 6.5)	21.0 (17.6, 25.0)	25.6 (13.5, 43.3)
Difficulty finding sexual partners	23.5 (18.0, 30.1)	44.4 (40.3, 48.6)	25.6 (14.7, 40.8)
Family did not care for you when you were sick	15.0 (11.1, 19.8)	24.5 (20.8, 28.7)	15.4 (6.2, 33.3)
Treated badly by health professionals	3.4 (1.9, 6.1)	16.6 (13.4, 20.4)	12.8 (5.4, 27.6)
Lost friends	5.8 (3.7, 8.9)	25.7 (21.7, 30.2)	20.5 (9.7, 38.2)
Disowned or neglected by family	9.9 (7.0, 13.8)	24.1 (20.0, 28.6)	15.4 (6.1, 33.8)
Experienced break-up of marriage or relationship	10.9 (7.8, 15.1)	28.7 (24.3, 33.7)	18.0 (7.9, 35.7)
Community (village) treated you like a social outcast	13.6 (10.0, 18.2)	27.6 (21.3, 32.2)	15.4 (6.2, 33.3)
Spouse/partner(s) became physically violent because they learned you had the virus	12.2 (8.6, 17.1)	25.0 (21.0, 29.5)	20.5 (9.5, 38.8)

### Social support

Frequencies of social support items are displayed in Table 3. Less than half (48.5%) of caregivers reporting that they have someone for all four scenarios. Although there was an association between marital status and social support (χ^2^ p-value <0.001), only 60.2% of caregivers who were married or living with a partner and included in the full analytical sample reported “yes” for all four questions.

**Table 3:** Caregiver social support.

Caregiver has someone…	Full analytical sample (n = 761) % (95% CI)	ART adherence sample (n = 289) % (95% CI)
To offer suggestions for how to deal with a problem	72.9 (69.5, 76.1)	75.1 (68.8, 80.5)
To help with daily chores if sick	75.8 (72.0, 79.3)	74.0 (67.8, 79.5)
Who shows love and affection	71.2 (67.3, 74.9)	66.1 (60.8, 71.0)
To do something enjoyable with	82.7 (79.7, 85.3)	78.5 (73.6, 82.7)

There is evidence that different types of social support may have different relationships with depression [33]. We conducted an exploratory analysis to examine each social support item separately in the multivariate linear regression for depressive symptom score, using all the previous controls. We found that tangible social support and social companionship support (having someone to show love and affection), but not emotional social support, were negatively associated with depressive symptoms.

### Relationship between depression, HIV status, and stigma

Table 4 displays the results for a multivariable linear regression of depressive symptom score on HIV-related stigma. The coefficient on HIV-related stigma represents anticipated stigma by those who report testing negative for HIV and is significantly associated with caregiver depressive symptoms. A 1-standard deviation (SD) increase in anticipated HIV-related anticipated stigma score is associated with a 0.19-SD higher depressive symptom score among caregivers who tested negative for HIV. Social support score was protective for depressive symptoms; a 1-SD increase of social support score was associated with 0.12-SD lower depressive symptoms score.

**Table 4:** Multivariable linear regression models for depressive symptoms by HIV status, HIV-stigma, and social support, (n = 761).

	Coefficient	p-value	95% Confidence Interval
HIV-related stigma	0.187	<0.001	(0.112, 0.262)
Social support score	-0.115	0.007	(-0.198, -0.033)
Economic insecurity	-0.066	0.449	(-0.237, 0.106)
Food insecurity	0.156	0.078	(-0.018, 0.330)
HIV status			
Living with HIV (self-reported)	0.355	<0.001	(0.179, 0.532)
Never tested for HIV	0.292	0.075	(-0.030, 0.614)
Stigma * HIV positive	0.302	0.028	(0.034, 0.570)
Stigma * never tested for HIV	0.127	0.287	(-0.109, 0.362)

Models control for caregiver sex, age, wealth, marital status, and completion of primary school (grade 8) or higher.

Standard errors clustered by census enumeration area.

Neither economic nor food insecurity were significantly associated with depressive symptoms. Self-report of testing positive for HIV was associated with a higher depressive symptoms score compared to self-report of testing negative for HIV. Never having been tested for HIV was associated with a higher depressive symptoms score compared to testing negative for HIV, though not significant at the 0.05 level. The significant interaction of living with HIV and HIV-related stigma score indicates that a higher perceived HIV-stigma score was associated with higher depressive symptoms scores. The interaction of never being HIV-tested and stigma was not significant at the 0.05 level.

### ART adherence

Nearly all (98%) caregivers who knew of their HIV positive status reported currently using antiretroviral medication. Of those on treatment, 90.8% reported never forgetting or missing a day of ART in the past 7 days. In a multivariable logistic regression of adherence on depressive symptoms (results in Table 5), a 1-SD increase in depressive symptom score was significantly associated with 36.1% lower odds of ART adherence (OR = 0.639, 95% CI = 0.435–0.940). Neither HIV-stigma score nor social support score was associated with ART adherence.

**Table 5:** Logistic regression predicting ART adherence among caregivers who reported testing positive for HIV and currently taking ART (n = 289).

	Odds Ratio	p-value	95% Conf. Interval	
Depressive symptoms score	0.639	0.023	0.435	0.940
Social support score	0.868	0.436	0.607	1.240
Stigma score	1.168	0.705	0.522	2.615
Wealth quintile				
Lower-middle	1.956	0.411	0.395	9.697
Middle	0.984	0.984	0.218	4.445
Upper-middle	0.808	0.763	0.202	3.233
Upper	0.695	0.623	0.163	2.969
Completed primary	2.893	0.243	0.487	17.184
Female	2.075	0.243	0.574	7.501
Age	1.010	0.694	0.959	1.065

Standard errors clustered by census enumeration area.

## Discussion

In this study, we found that depressive symptoms were prevalent among caregivers of children living in vulnerable households in the Malawian context, and more common among those reporting to have tested positive for HIV. This finding is aligned with other studies that have found that individuals living with HIV are more likely to have depressive symptoms compared to individuals who are HIV-negative [34]. Considering poor mental health outcomes among caregivers may negatively affect the health and development of their children [16–18], identifying pathways to resilience for caregivers experiencing depressive symptoms would improve both caregiver and child wellbeing.

Our results indicate that social support may be protective of depressive symptoms among caregivers of children living in vulnerable households. While mostly from developed countries, studies examining the relationship between social support and depression show a consistent association [35]. Given that households in our study are faced with at least one vulnerability (food insecurity, economic insecurity, and/or chronic illness in the household), it is not surprising that tangible support has a strong negative association with depressive symptoms. Having someone to help with daily chores, which may include cooking and caring for children, when the caregiver is sick may guarantee that basic physical needs can be met, and some level of companionship potentially provided. For caregivers living with HIV, tangible support may mean that they have someone who is aware of their HIV status and is willing to provide physical assistance. The fact that social companionship support was significantly associated with lower depressive symptoms when also controlling for marital status indicates that social support is not limited to caregivers with spouses. It is important to acknowledge the reciprocal relationship of social support with depression, as individuals may withdraw from social ties and isolate themselves. As this study cannot comment on the causality or direction of this relationship, more empirical research investigating the biopsychosocial mechanisms behind these relationships in the Malawian context is needed.

We found that perceived HIV-related stigma was associated with higher depression scores among those who reported having HIV, which is consistent with previous evidence from the region [36]. Anticipated HIV-stigma was also associated with depressive symptoms among caregivers who reported testing negative for HIV. There could be many explanations for why the association of anticipated stigma and depressive symptoms exists. Caregivers reporting high anticipated stigma scores may feel that their community is not tolerant or supportive, which may affect their mental health even if they are not experiencing the stigma firsthand. Caregivers may have even experienced stigma from something other than HIV, including their mental disturbance, further affecting their feelings of connectedness and self-worth. Further, although they may have had a negative HIV test result in the past, only about half (51.3%) of those who have tested negative for HIV were tested for HIV in the past 6 months. It is possible that their perceived risk of contracting HIV is high, and some among this group may believe they have HIV or be very concerned of contracting HIV. This worry, along with internalized stigma, may explain the relationship between anticipated stigma and depressive symptoms.

ART nonadherence increases the likelihood that treatment will fail, that the individual will develop ART resistance and s/he will continue to transmit HIV [37–39]. We found that depressive symptoms, but not HIV-related stigma or social support, were associated with ART nonadherence among caregivers who reported living with HIV. The significant and negative relationship between depressive symptoms and ART adherence indicates that those who are depressed may be experiencing an internalized obstacle to adherence, stemming from the depressive symptoms. Studies have shown that depressed individuals are more likely to be nonadherent to medical treatment, including ART, and this relationship is consistent across contexts and time [40]. It is postulated that because mood disorders such as depression weaken cognitive focus, energy, and motivation, they might impair an individual’s willingness and ability to adhere to treatment [41]. Further, feelings of hopelessness and social isolation may contribute to a disbelief in the effectiveness of the treatment and preclude the individual from spending time with those who might support compliance.

Our results have implications for both caregiver and child wellbeing. Households that are already vulnerable due to economic insecurity, food insecurity, and/or the strain of an adult member having a chronic illness may face additional obstacles due to a caregiver struggling with depressive symptoms and potentially facing ART failure. These obstacles may pose threats to a child’s wellbeing as caregivers may not be able to physically or emotionally support children in their households. As the caregivers in our sample appear to have been able to access primary HIV care to obtain ART, this suggests a good venue for integration of mental health services. A recent study in central Malawi piloted a capacity building program that integrated depression screening and treatment into HIV primary care [42]. The success of this program demonstrated that this integration is achievable in the Malawi context. Bringing this intervention to scale may help alleviate the prevalence of depression among caregivers in vulnerable households living with HIV.

Limitations to this study must be considered. Because this study was cross-sectional and the nature of the relationships between stigma, HIV-status, depression and ART adherence are complex, causal inferences cannot be drawn and the direction of the associations cannot be inferred. Many of these associations could plausibly be bidirectional. For example, the relationships between depression and HIV status, HIV-stigma and social support can all function in both directions. Additionally, HIV status in this study was limited to self-report and not confirmed with testing. As a consequence of using self-reported status, caregivers reporting to be HIV-negative may be false negatives due to lack of a current test result. Self-report of HIV-positive status is likely more valid. Self-report of positive HIV status has been shown to be a valid measure of true HIV status in similar settings in sub-Saharan Africa (positive predictive value 94%) [43]. Furthermore, those who are on ART are more likely to report their status as HIV-positive. Additionally, since we were not able to test for HIV, some of the caregivers were never tested. To account for this, we included those caregivers in our model, but created a separate category for those never tested to see if results differed compared to those with a test result. Furthermore, our measure of adherence is relatively simple, as our survey did not ask how many prescribed pills had been missed. Therefore, we only considered those who reported not missing a dose of medication on any day in the last seven days as adherent (100% adherence). Other factors such as CD4+ count, viral load, comorbid psychiatric conditions that may also associate with depressive symptoms, and perceived risk of HIV were not captured in our study or tested.

Our study provides a unique perspective to the field as one of the few studies in Malawi to assess the relationship between depressive symptoms, HIV status and ART adherence among caregivers of children living in vulnerable households. Our results are programmatically relevant as they provide evidence of the need to screen people living with HIV for depression during the course of their care and treatment and provide supportive interventions for those with depressive symptoms in order to support their ART adherence.

## Supporting information

S1 Table Survey questions for variables of interest. (DOCX)
