# Correction: Depressive symptoms, HIV-related stigma and ART adherence among caregivers of children in vulnerable households in rural southern Malawi

**DOI:** 10.1371/journal.pone.0316523

**Published:** 2024-12-26

**Authors:** Kathryn L. Spielman, Erica Soler-Hampejsek, Adamson S. Muula, Lyson Tenthani, Paul C. Hewett

The following information is missing from the Competing Interests statement: This article was prepared while Paul C. Hewett was employed at The Population Council. The opinions expressed in this article are the author’s own and do not reflect the view of the National Institutes of Health, the Department of Health and Human Services, or the United States government.

## References

[1] Spielman KL, Soler-Hampejsek E, Muula AS, Tenthani L, Hewett PC (2021) Depressive symptoms, HIV-related stigma and ART adherence among caregivers of children in vulnerable households in rural southern Malawi. PLOS ONE 16(3): e0247974. 10.1371/journal.pone.0247974 33667258 PMC7935323

