# Supplementary material for: Depressive symptoms, HIV-related stigma and ART adherence among caregivers of children in vulnerable households in rural southern Malawi
Source: PLoS One. 2021 Mar 5;16(3):e0247974. doi: 10.1371/journal.pone.0247974 (PMC7935323; doi:10.1371/journal.pone.0247974)
Supplement: S1 Table — (DOCX) [file pone.0247974.s001.docx]

|  | Survey items | Survey instrument and  source |
| --- | --- | --- |
| Depressive symptoms z-score  Unstandardized  score range: 0-10  Alpha: 0.74 | 1. In the past 4 weeks, how often did you take little interest or pleasure in doing things that you enjoy? (0 point if ‘never,’ 1 point if ‘sometimes,’ 2 points if ‘often’) 2. In the past 4 weeks, how often did you feel down, depressed or hopeless? (0 point if ‘never,’ 1 point if ‘sometimes,’ 2 points if ‘often’) 3. In the past 4 weeks, how often did you have trouble falling or staying asleep because you are restless? (0 point if ‘never,’ 1 point if ‘sometimes,’ 2 points if ‘often’) 4. In the past 4 weeks, how often did you sleep too much of for too long? (0 point if ‘never,’ 1 point if ‘sometimes,’ 2 points if ‘often’) 5. In the past 4 weeks, how often were you irritable or easily annoyed? (0 point if ‘never,’ 1 point if ‘sometimes,’ 2 points if ‘often’) | OVC Caregiver survey  Source: Measure Evaluation OVC |
|  |  |  |
| Social support z-score  Unstandardized  score range: 0-4  Alpha: 0.63 | 1. Do you have someone in your life to turn to for suggestions about how to deal with a personal problem? (1 point if ‘yes’) 2. Do you have someone in your life to help with daily chores if you were sick? (1 point if ‘yes’) 3. Do you have someone in your life that shows you love and affection? (1 point if ‘yes’) 4. Do you have someone in your life to do something enjoyable with? (1 point if ‘yes’) | OVC Caregiver survey  Source: Measure Evaluation OVC |
| HIV-related stigma |  |  |
| Anticipated HIV-related stigma  Unstandardized  score range: 0-10  Alpha:0.90 | If you tested positive for HIV and told others about your status, how likely is it that:   1. You would lose your job/livelihood. (1 point if ‘very likely’ or ‘somewhat likely’) 2. You would be treated badly at work or school. (1 point if ‘very likely’ or ‘somewhat likely’) 3. You would have difficulty finding sexual partners in the future. (1 point if ‘very likely’ or ‘somewhat likely’) 4. Your family would not care for you if you became sick. (1 point if ‘very likely’ or ‘somewhat likely’) 5. You would be treated badly by health professionals. (1 point if ‘very likely’ or ‘somewhat likely’) 6. You would lose your friends. (1 point if ‘very likely’ or ‘somewhat likely’) 7. You would be disowned from or neglected by your family. (1 point if ‘very likely’ or ‘somewhat likely’) 8. You would experience a break-up of your marriage or relationship. (1 point if ‘very likely’ or ‘somewhat likely’) 9. Your community (village) would treat you like a social outcast. (1 point if ‘very likely’ or ‘somewhat likely’) 10. Your spouse/partner(s) would be physically violent. (1 point if ‘very likely’ or ‘somewhat likely’) | OVC Caregiver survey  Source:  Adapted from Link-Up Study |
| Perceived HIV-related stigma  Unstandardized  score range: 0-10  Alpha: 0.74 | 1. You lost your job/livelihood. (1 point if ‘yes’) 2. You were treated badly at work or school. (1 point if ‘yes’) 3. You have had difficulty finding sexual partners. (1 point if ‘yes’) 4. Your family did not care for you if you were sick. (1 point if ‘yes’) 5. You were treated badly by health professionals. 6. You lost friends. (1 point if ‘yes’) 7. You were disowned from or neglected by your family. (1 point if ‘yes’) 8. You experienced a break-up of your marriage or relationship. (1 point if ‘yes’) 9. Your community (village) treated you like a social outcast. (1 point if ‘yes’) 10. Your spouse/partner(s) became physically violent because they learned your HIV status. (1 point if ‘yes’) | OVC Caregiver survey  Source:  Adapted from Link-Up Study |
| ART adherence  (0/1) | In the last 7 days, on how many days did you forget or miss taking your medication? (1 if 0 days) | OVC Caregiver survey  Source: adapted from Malawi Population-based HIV Impact Assessment |
|  |  |  |
| Food insecurity  (0/1) | 1 if household head reports ‘Often (>10 times in past 4 weeks)’ to any of the following questions:   1. In the past 4 weeks was there ever no food to eat of any kind in your house because of lack of resources to get food? 2. In the past 4 weeks, did you or any household member go to sleep at night hungry because there was not enough food? 3. In the past 4 weeks, did you or any household member go a whole day and night without eating anything at all because there was not enough food? | Household screening  Source:  Measure Evaluation Caregiver Questionnaire v. 1.4 |
|  |  |  |
| Economic insecurity  (0/1) | 1 if household head reports ‘no’ to (a) AND (EITHER ‘yes’ to (b) OR ‘no’ to (c))   1. If there was an unexpected urgent household expense today (e.g. emergency medical expense including transport to a facility or house repair), would your household be able to pay for that expense? 2. Does the household head or his/her spouse have any form of disability or illness that prevents him/her from engaging in work? 3. In the past 6 months, has anyone in this household had consistent formal or informal work that generated money for the household? | Household screening  Source:  Measure Evaluation Caregiver Questionnaire v. 1.4 |
|  |  |  |
| Chronic illness  (0/1) | 1 if household head reports > 1 for any of the following:   1. How many of household members aged 18 and older have been very sick for at least 3 months during the past 12 months, that is too sick to work or do normal activities? 2. How many household members (of any age) are taking any long-term medicine or medication that they expect to take for the rest of their life? 3. Has anyone in this household been tested and told that they have HIV/AIDS? (1 if ‘yes’) | Household screening |
